# Limbic Network and Papez Circuit Involvement in ALS: Imaging and Clinical Profiles in GGGGCC Hexanucleotide Carriers in *C9orf72* and *C9orf72*-Negative Patients

**DOI:** 10.3390/biology13070504

**Published:** 2024-07-06

**Authors:** Foteini Christidi, Jana Kleinerova, Ee Ling Tan, Siobhan Delaney, Asya Tacheva, Jennifer C. Hengeveld, Mark A. Doherty, Russell L. McLaughlin, Orla Hardiman, We Fong Siah, Kai Ming Chang, Jasmin Lope, Peter Bede

**Affiliations:** 1 Computational Neuroimaging Group (CNG), School of Medicine, Trinity College Dublin, D08 W9RT Dublin, Ireland; 2 Department of Neurology, St James’s Hospital, D08 KC95 Dublin, Ireland; 3 Smurfit Institute of Genetics, Trinity College Dublin, D08 W9RT Dublin, Ireland

**Keywords:** amyotrophic lateral sclerosis, motor neuron disease, neuropsychology, cognition, MRI, neuroimaging, biomarker, C9orf72, presymptomatic, genotype

## Abstract

**Simple Summary:**

Amyotrophic lateral sclerosis (ALS) is the most common form of Motor Neuron Disease and is traditionally associated with motor cortex, brainstem and spinal cord degeneration. Neuropsychological deficits are also increasingly recognized in ALS and have considerable clinical ramifications, but existing studies of cognitive impairment in ALS have primarily focused on cortical frontotemporal disease burden. The aim of this study is the comprehensive assessment of the grey and white matter components of limbic networks in a large cohort of patients with ALS, stratified for the *C9orf72* genotype. Our MRI analyses reveal that the cortical, subcortical and white matter components of limbic circuits are not only affected in *C9orf72*-positive patients, but also in those who test negative for this genetic variant. Our radiological findings are consistent with previous neuropsychological observations and highlight the importance of comprehensive neuropsychological testing in ALS, irrespective of the underlying genotype. Cognitive impairment in ALS has widespread practical implications, including compliance with assistive devices, participation in clinical trials, and it has been associated with increased caregiver burden and is widely regarded as an adverse prognostic indicator. Our data provide radiological evidence of widespread limbic degeneration in ALS, which is particularly severe in *C9orf72* mutation carriers.

**Abstract:**

**Background**: While frontotemporal involvement is increasingly recognized in Amyotrophic lateral sclerosis (ALS), the degeneration of limbic networks remains poorly characterized, despite growing evidence of amnestic deficits, impaired emotional processing and deficits in social cognition. **Methods**: A prospective neuroimaging study was conducted with 204 individuals with ALS and 111 healthy controls. Patients were stratified for hexanucleotide expansion status in *C9orf72*. A deep-learning-based segmentation approach was implemented to segment the nucleus accumbens, hypothalamus, fornix, mammillary body, basal forebrain and septal nuclei. The cortical, subcortical and white matter components of the Papez circuit were also systematically evaluated. **Results**: Hexanucleotide repeat expansion carriers exhibited bilateral amygdala, hypothalamus and nucleus accumbens atrophy, and C9orf72 negative patients showed bilateral basal forebrain volume reductions compared to controls. Both patient groups showed left rostral anterior cingulate atrophy, left entorhinal cortex thinning and cingulum and fornix alterations, irrespective of the genotype. Fornix, cingulum, posterior cingulate, nucleus accumbens, amygdala and hypothalamus degeneration was more marked in *C9orf72*-positive ALS patients. **Conclusions**: Our results highlighted that mesial temporal and parasagittal subcortical degeneration is not unique to *C9orf72* carriers. Our radiological findings were consistent with neuropsychological observations and highlighted the importance of comprehensive neuropsychological testing in ALS, irrespective of the underlying genotype.

## 1. Introduction

Amyotrophic lateral sclerosis is primarily associated with relentless motor neuron degeneration manifesting in progressive motor disability, bulbar dysfunction and respiratory insufficiency [1]. Accordingly, neuroimaging studies have traditionally primarily focused on the motor cortex, corticospinal tracts, brainstem and spinal cord [2,3,4,5]. Clinical case series, however, have long highlighted coexisting frontotemporal, extrapyramidal subcortical, sensory and cerebellar dysfunction [6,7,8,9]. The substrate of extra-motor manifestations have been characterized by nuanced post mortem studies and have also been evaluated in vivo by comprehensive neuroimaging studies [10,11,12,13]. While neuroimaging studies focusing on extra-motor involvement in ALS have consistently captured hippocampal [14], amygdalar [15], dorsolateral frontal lobe [8], orbitofrontal [16], temporal lobe [2], insular [17] and Broca’s area involvement [18], fornix, limbic nuclei and the integrity of the Papez circuit have been characterized much less well [19,20,21,22]. Subcortical grey matter studies in amyotrophic lateral sclerosis invariably detect volume reductions, but subcortical structures are typically evaluated as a whole, i.e., the atrophy of the entire thalamus or entire amygdala, as opposed to the evaluation of specific nuclei. This is important as these nuclei mediate specific cortico–basal and cortico–cortical circuits with distinct neuropsychological, motor and sensory functions [23,24,25]. Subsequent to the incremental characterization of both motor system and frontotemporal pathology in ALS, there have been emerging reports of selective cerebellar involvement in ALS [26,27,28,29,30]. Beyond gait impairment and changes in dexterity, cerebellar dysfunction in ALS may also contribute to dysarthria, dysphagia and abnormal respiratory patterns, and are likely to have specific cognitive and behavioural sequelae also.

**Figure 1:**
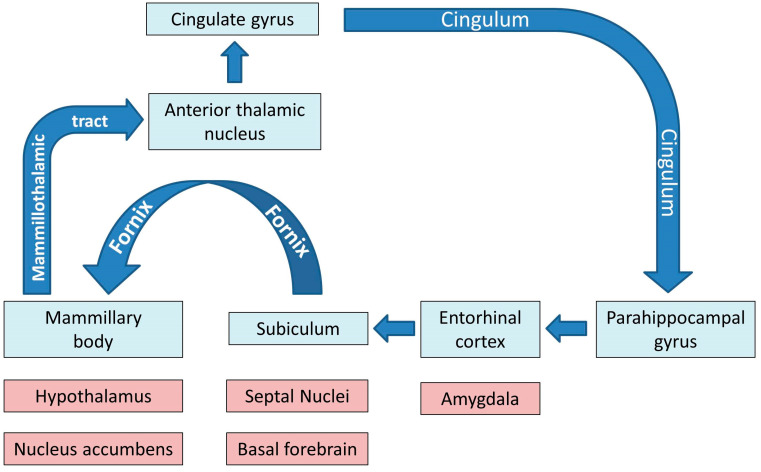
The main grey and white matter components of limbic networks; the blue colour indicates the main structures of the Papez circuit.

Physiologically, the limbic system and the Papez circuit (Figure 1) mediate a number of crucial functions such as motivation, emotional regulation, information registration and spatial memory, etc. [31], therefore the dysfunction of these networks in ALS has important practical ramifications. While clinical trials continue to focus solely on motor function, mobility, bulbar function, respiratory measures and composite functional rating scale scores as their main monitoring and outcome measures, the practical impact of cognitive and behavioural impairment should not be underestimated [32,33]. Neuropsychological deficits in ALS are thought to have survival ramifications, impact on caregiver burden, affect compliance with assistive devices, end-of-life decisions, adherence to therapy and participation in clinical trials [34,35].

Cognitive profiles in ALS have been traditionally primarily associated with executive dysfunction, verbal fluency deficits and behavioural impairment, but memory impairment, language deficits and apathy are increasingly recognized facets of neurocognitive change in ALS [14,36,37,38,39,40,41,42,43]. More recently, a multitude of studies have highlighted difficulties in social cognition and theory of mind deficits, which again may impact on clinical care. Pursuing direct correlations between focal imaging measures and functional performance has long been regarded as contentious, but more recently the altering and buffering effects of education and cognitive reserve are also recognized as important modifiers of clinical performance [44,45,46,47].

In light of the clinical relevance of limbic network dysfunction and the relative paucity of imaging studies specifically focusing on these structures, we conducted a prospective, multimodal imaging study of limbic disease burden in ALS in a large cohort of patients with ALS, stratified for the *C9orf72* hexanucleotide repeat expansions. Our main objectives were the comprehensive assessment of both the white and grey matter components of these circuits, and to assess their degeneration in sporadic patients with ALS and those carrying the *C9orf72* repeat expansion. Based on clinical observations, we hypothesized that those carrying the repeat expansion would exhibit a more marked degeneration of these structures, but we also hypothesized that *C9orf72*-negative individuals with ALS would also suffer from limbic network dysfunction.

## 2. Materials and Methods

### 2.1. Ethics Approval

This research project was approved by the Beaumont Ethics Medical Research Committee, Beaumont Hospital, Dublin (REC reference: 08/90), and all participants gave informed consent to participate.

### 2.2. Participants

A prospective multimodal neuroimaging study was conducted with 315 participants; 204 individuals with ALS and 111 healthy controls. Patients with ALS were diagnosed according to the revised El Escorial criteria and had “probable” or “definite” ALS. Healthy controls were unrelated to the patients and had no known first degree relatives with neurodegenerative conditions. Exclusion criteria prior to imaging for both patients and healthy controls (HC) included prior brain surgery, known cerebral infarcts or haemorrhages, traumatic brain injuries, known hydrocephalus or brain tumours, comorbid psychiatric conditions, multiple sclerosis, or systemic conditions such as malignancies, vasculitis, or HIV. Potential participants were also screened for suitability for MRI scanning, therefore individuals with pacemakers, aneurysm clips, orbital metallic fragments, or severe self-declared claustrophobia were not enrolled. A smoking history, alcohol consumption, a detailed family history, and medical and surgical history were taken from each potential participant prior to enrolment in the study. In this particular study, only ALS patients with comprehensive genetic screening were included (see details below) and patients with neuroimaging data but no genetic screening were excluded. All patients and controls underwent neuroimaging with the same MRI protocol using uniform scanner settings (see details below) on the same scanner. Only participants who had a full data set including both 3D T1-weighted images and diffusion tensor imaging (DTI) data were included, and participants with incomplete data, i.e., missing T1 or DTI, were excluded. Patients and controls with incidental cerebral findings such as meningioma, hydrocephalus, demyelination, arachnoid cysts, or prior infarcts were also excluded. In patients who had multiple follow-up imaging, their first data set was included. Family history, handedness, age, sex and education were systemically recorded from all study participants. The recorded demographic data were subsequently used as covariates in statistical models (see details below).

### 2.3. Clinical Assessments

Site of onset (spinal/bulbar), revised ALS functional rating scale (ALSFRS-r) scores [48] and symptom duration were recorded in each individual with ALS. A total of 159 out of 204 patients had a brief screening cognitive assessment with the Edinburgh Cognitive and Behavioural ALS Screen (ECAS) (REF) administered within one week of their MRI scan. The ECAS is a validated neuropsychological screening instrument that assesses the language, verbal fluency, executive, memory and visuospatial domains [49]. It has been comprehensively validated worldwide, including by the Irish population, and local normative values have been generated [50]. A subset of patients also took the Penn UMN scale [51], HADS [52] and Emotional Lability Questionnaire (ELQ) [53], and had a comprehensive sensory [25] and cerebellar assessment [27], but these data were not specifically explored in this particular study.

### 2.4. Genetics

Each patient with ALS was screened for both a range of ALS-associated genetic variants and for hexanucleotide repeat expansions in *C9orf72*. Repeat-primed polymerase chain reaction (PCR) was used to screen for intronic GGGGCC repeat expansion in *C9orf72*, as described previously [54]. GeneMapper version 4.0 was used to visualise capillary electrophoresis outcomes, and 30 or more repeats were considered *C9orf72*-positive. All participating patients were also screened for a panel of protein-altering, exonic, or splice-site variants in 32 genes linked to ALS in the ALS online database (ALSod) [55], including *ALS2*, *ANG*, *ATXN2*, *CHCHD10*, *CHMP2B*, *DAO*, *DCTN1*, *ELP3*, *ERBB4*, *FIG4*, *FUS*, *HNRNPA1*, *MATR3*, *NEFH*, *NEK1*, *OPTN*, *PFN1*, *PRPH*, *SARM1*, *SETX*, *SIGMAR1*, *SOD1*, *SPAST*, *SPG11*, *SQSTM1*, *TAF15*, *TARDBP*, *TBK1*, *UNC13A*, *UBQLN2*, *VAPB* and *VCP.* Either whole-genome sequence data [56] or targeted DNA sequence data [55] were utilised. Following quality control, sequence data were aligned to the GRCh37 reference genome, and variants were annotated and analysed using cutadapt V.1.9.1, SAMtools V1.7, Picard V.2.15.0 (http://broadinstitute.github.io/picard/ accessed on 5 July 2024), Plink V.1.9, R V.3.2.3 (http://www.r-project.org/ accessed on 5 July 2024), SnpEff V.4.3 and Gemini V.0.20.1.

### 2.5. Neuroimaging

#### 2.5.1. Data Acquisition

MRI data of all participants were acquired on the same 3 Tesla Philips Achieva platform. Patients were screened for incidental neurovascular or neuroinflammatory findings by fluid-attenuated inversion recovery (FLAIR) imaging. FLAIR images were recorded axially, implementing an Inversion Recovery Turbo Spin Echo (IR-TSE) sequence with the following settings: TR/TE = 11,000/125 ms, TI = 2800 ms, FOV = 230 × 183 × 150 mm, spatial resolution = 0.65 × 0.87 × 4 mm. Two main raw input data sets were analysed quantitatively in this study: 3D structural T1-weighted (T1w) images and diffusion-weighted images (DWI). T1-weighted data were acquired with a 3D Inversion Recovery-prepared Spoiled Gradient Recalled echo (IR-SPGR) sequence with a 1mm isotropic voxel resolution (VR), a field-of-view (FOV) of 256 × 256 × 160 mm, 160 sagittal slices with no interslice gap, flip angle (FA) = 8°, SENSE factor = 1.5, TR/TE = 8.5/3.9 ms and TI =1060 ms. DTI data were acquired with a spin-echo echo planar imaging (SE-EPI) pulse-sequence implementing a 32-direction Stejskal-Tanner diffusion encoding scheme; FOV = 245 × 245 × 150 mm, 60 axial slices with no interslice gaps, FA = 90°, VR = 2.5 mm isotropic, SENSE factor = 2.5, TR/TE = 7639/59 ms, dynamic stabilisation and spectral presaturation with inversion recovery (SPIR) fat suppression.

#### 2.5.2. Data Analysis: Segmentation and Volumetric Analysis

**Figure 2:**
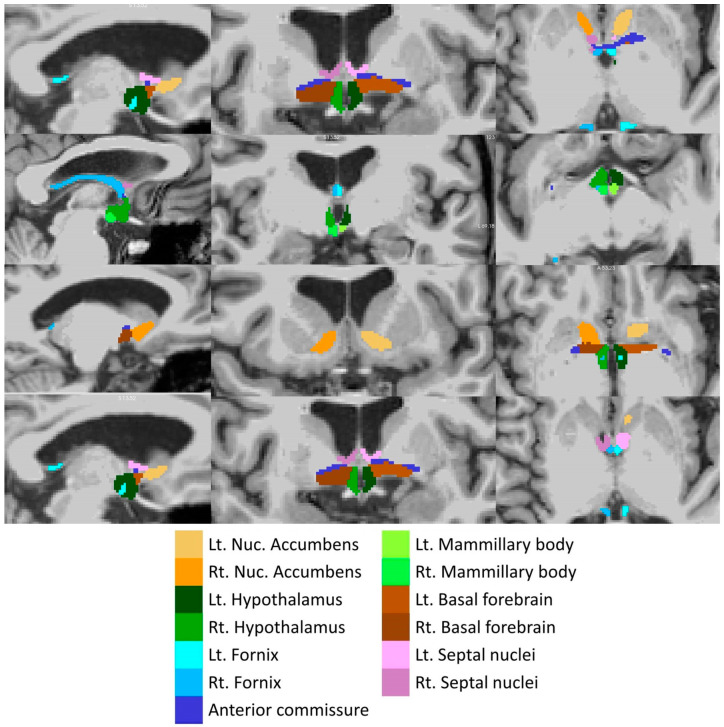
Deep-learning based subcortical segmentation of limbic structures, Lt: Left, Rt: Right.

Total intracranial volumes (TIV) were estimated in FreeSurfer, implementing Buckner et al.’s approach [57]. The volumes of the left and right amygdala were retrieved from the basic pre-processing output of FreeSurfer. The subcortical limbic segmentation toolbox [58] was implemented to segment the nucleus accumbens, hypothalamus, fornix, mammillary body, basal forebrain and septal nucleus in both hemispheres separately (Figure 2). The toolbox relies on a U-Net deep-learning architecture with spatial, intensity, contrast and noise augmentation trained on 39 manually labelled data sets and extensively validated with excellent true positive rates, false discovery rates and manual–automatic volume correlations [58]. The pipeline was implemented with single T1-weighted inputs, and segmentation accuracy was individually verified in all subjects, in both patients and controls, using “freeview”. The thalamus was segmented into 25 sub-regions by a Bayesian inference pipeline implementing a probabilistic atlas developed using histological data [59], and the volumes of the left and right anterior thalamic nuclei were retrieved. The hippocampus was parcellated into cytologically-defined subfields implementing the hippocampal segmentation stream [60] of FreeSurfer to generate volumetric estimates for the left and right subiculum.

#### 2.5.3. Data Analysis: Cortical Thickness Analysis

The pre-processing and anatomical reconstruction pipeline of the FreeSurfer image analysis suite [61] was utilised with the following standard steps: non-parametric non-uniform intensity normalisation, affine registration to the MNI305 atlas, intensity normalisation, skull striping, automatic subcortical segmentation, linear volumetric registration, neck removal, tessellation of the grey matter–white matter boundary, surface smoothing, inflation to minimise metric distortion and automated topology correction [62]. Eight processing cores were utilised in parallel, given the considerable computational demands and time of the recon-all pipeline. The cortical labels of the Desikan–Killiany atlas [63] were used to estimate average cortical thickness values from the entorhinal, parahippocampal, caudal anterior cingulate, posterior cingulate and rostral anterior cingulate gyri separately in the left and right hemispheres.

#### 2.5.4. Data Analysis: DTI Analysis

**Figure 3:**
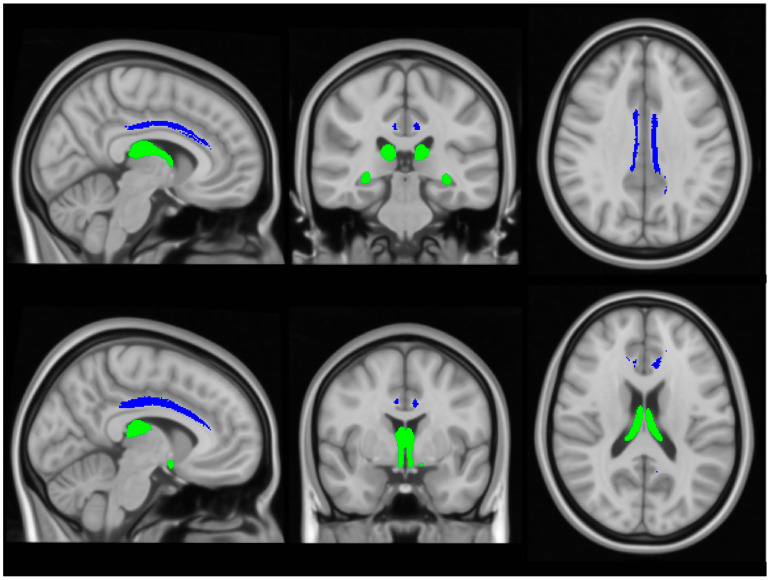
The assessment of white matter integrity in the fornix (green) and cingulum (blue), based on diffusivity measures.

Input raw diffusion tensor imaging data were pre-processed using tools of the FMRIB’s software library (FSL) (https://fsl.fmrib.ox.ac.uk/fsl/docs/#/ accessed on 5 July 2024). DTI data were eddy current-corrected, skull stripped, and a tensor model was fitted to create fractional anisotropy (FA), axial diffusivity (AD) and radial diffusivity (RD) mpas. FSL’s tract-based statistics (TBSS) module was then utilised for non-linear registration, skeletonisation and the creation of a mean FA mask. Each participant’s individual FA, AD and RD images were then merged into 4-dimentional (4D) AD, FA and RD image files. Diffusivity metrics were retrieved from the four (AD, FA, RD) concatenated diffusivity files using the FMRIB fornix label [64] and the cingulum labels of the ICBM-DTI-81 white-matter atlas [65] in the two hemispheres separately (Figure 3). The Fornix_FMRIB_FA1mm template [64] is derived from probabilistic tractography pathways of the fornix in 49 adults registered to FMRIB58_FA standard-space and averaged [64].

### 2.6. Statistical Analysis

Normality assumptions on MRI-dependent variables were verified before parametric statistics were implemented. Differences in age, education and sex distribution across HC and ALS subgroups were examined with one-way analysis of variance and χ2-test, respectively. To test the effect of group on subcortical volumes, cortical thickness and WM integrity indices, multivariate analyses of covariance (MANCOVAs) were conducted using the MRI metrics as dependent variables, group membership (HC, ALS-neg, ALS-pos) as an independent factor, and age, sex, handedness and TIV (only for the volumetric analysis) as covariates. In case of a significant multivariate omnibus test, post-hoc comparisons were conducted. Post-hoc contrasts were considered significant at *p* < 0.05, following Bonferroni corrections for multiple comparisons to reduce Type I error. To examine the contribution of neuroimaging metrics on core limbic system cognitive function, i.e., memory, we conducted regression analysis, including ECAS-total memory score as a dependent variable, and age, sex, education and neuroimaging metrics as independent (confounding or predictor) variables. To avoid clinically irrelevant neuroimaging contributions to memory performance, only neuroimaging metrics with a significant main effect on previous MANCOVAs were entered as predictors. Statistical analyses were conducted using IBM SPSS v. 29.

## 3. Results

### 3.1. Demographic and Clinical Profile of Study Participants

The three groups were matched for age and education, but not for sex and handedness. The two patient groups were matched for site onset, symptom duration and functional disability (Table 1). Regarding cognitive status, the two patient groups differed in ECAS-Memory, with C9+ALS showing worse performance compared to C9-ALS (*p* = 0.047). Apart from the *C9orf72* repeat expansion, no patient carried a pathogenic or likely pathogenic variant in any of the 32 genes that were analyzed.

### 3.2. Volumetric Analysis

**Figure 4:**
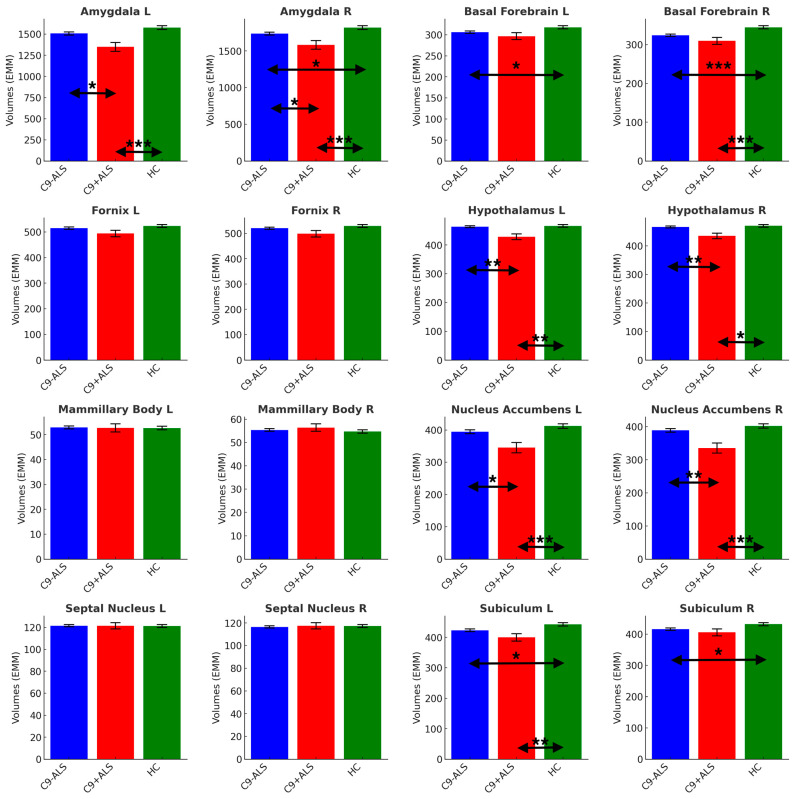
The limbic volumetric profiles of the study groups. Estimated marginal means (EMM) were calculated, with age, sex, handedness and TIV as covariates. Each measure is shown with error bars representing the standard error (SE). Horizontal arrows indicate statistically significant group differences with the following significance thresholds * *p* < 0.05, ** *p* <= 0.005, *** *p* <= 0.001.

A significant main effect of group was detected in volumetric analysis (Pillai’s Trace = 0.180; F = 1.819; *p* = 004). Limbic structures’ segmentation revealed study group-specific volumetric profiles (Figure 4).

Significant atrophy was identified in bilateral amygdala (L and R: C9+ALS < HC, C9-ALS > C9+ALS; R: C9-ALS < HC), basal forebrain (L and R: C9-ALS < HC; R: C9+ALS < HC), hypothalamus (L and R: C9+ALS < HC; C9-ALS > C9+ALS), nucleus accumbens (L and R: C9+ALS < HC; C9-ALS > C9+ALS), and subiculum (L: C9-ALS < HC, C9+ALS < HC; R: C9-ALS < HC) (Table 2).

### 3.3. Cortical Thickness Analysis

**Figure 5:**
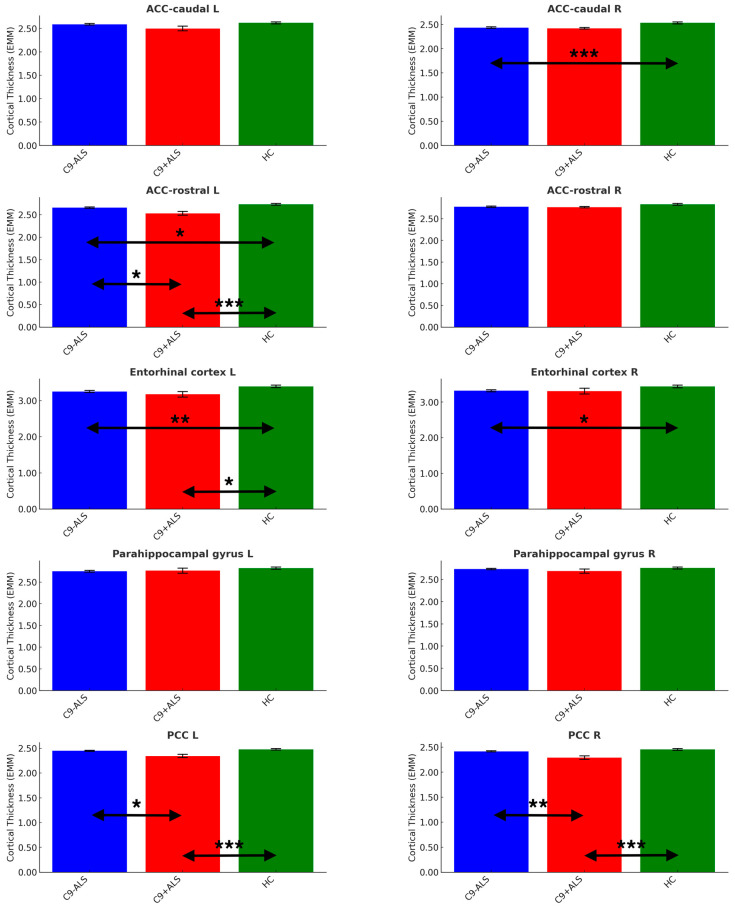
The limbic cortical thickness profiles of the study groups. Estimated marginal means (EMM) were calculated, with age, sex and handedness as covariates. Each measure is shown with error bars representing the standard error (SE). Horizontal arrows indicate statistically significant group differences with the following significance thresholds * *p* < 0.05, ** *p* <= 0.005, *** *p* <= 0.001.

A significant main effect of group was identified in cortical thickness analyses (Pillai’s Trace = 0.159; F = 2.596; *p* < 0.001). Cortical thickness analyses revealed considerable cortical changes in both ALS groups (Figure 5).

Significant differences were noted in ACC (caudate R: C9-ALS < HC; rostral L: C9-ALS < HC, C9+ALS < HC, C9-ALS > C9+ALS), PCC (L and R: C9+ALS < HC; C9-ALS > C9+ALS) and entorhinal cortex (L: C9-ALS < HC; C9+ALS < HC; R: C9-ALS < HC) (Table 3).

### 3.4. DTI Analysis

**Figure 6:**
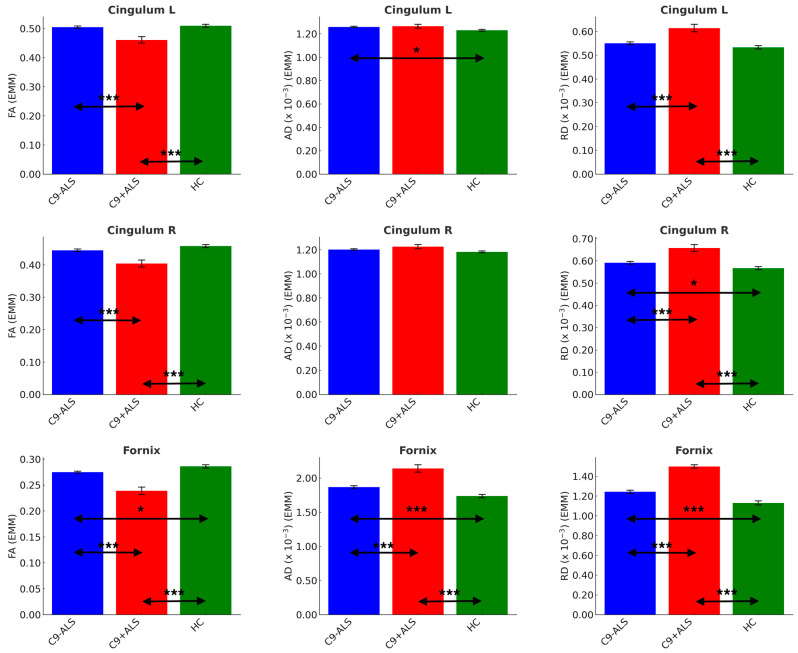
The limbic diffusivity profiles study group. Estimated marginal means (EMM) were calculated with age, sex and handedness as covariates. Each measure is shown with error bars representing the standard error (SE). Horizontal arrows indicate statistically significant group differences with the following significance thresholds * *p* < 0.05, *** *p* <= 0.001.

A significant main effect of group was also captured in DTI analyses (Pillai’s Trace = 0.223; F = 4.219; *p* < 0.001). DTI analyses revealed symmetrical involvement in limbic white matter tracts (Figure 6).

Significant inter-group differences were detected in cingulum FA (L and R: C9+ALS < HC; C9-ALS > C9+ALS), AD (L: C9-ALS > HC) and RD (L and R: C9+ALS > HC; C9-ALS < C9+ALS; R: C9-ALS > HC), as well as in all DTI metrics of fornix (FA: C9-ALS < HC, C9+ALS < HC, C9-ALS > C9+ALS; AD and RD: C9-ALS > HC, C9+ALS > HC, C9-ALS < C9+ALS) (Table 4).

### 3.5. Regression Analysis Models

Within the C9-ALS group, the overall model reached significance, F = 13.822; *p* < 0.001; adjusted R^2^ = 0.124. Volume of the left hypothalamus (b = 0.019; *p* ≤ 0.001) and cortical thickness of the left entorhinal gyrus (b = 1.526; *p* = 0.020) emerged as significant predictors, i.e., higher left hypothalamic volume and higher left entorhinal cortical thickness predicted superior memory performance of C9-ALS patients. Within the C9+ALS group, the overall final model reached significance, F = 9.199; *p* = 0.007; adjusted R^2^ = 0.281. Volume of the left nucleus accumbens (b = 0.036; *p* = 0.007) emerged as a significant predictor of the model; the higher the left nucleus accumbens volume, the better the ECAS memory performance of C9+ALS patients.

## 4. Discussion

Our data demonstrated that hexanucleotide repeat *C9orf72* expansion carriers exhibited bilateral amygdala, hypothalamus and nucleus accumbens atrophy, but *C9orf72*-negative patients also showed bilateral basal forebrain volume reductions compared to controls. With respect to Papez circuit degeneration, both patient groups showed left rostral anterior cingulate atrophy, left entorhinal cortex thinning, and cingulum and fornix diffusivity alterations compared to controls, irrespective of the genotype. Fornix and cingulum diffusivity changes, bilateral posterior–cingulate–context–thinning, left rostral anterior–cingulate–thickness–loss, nucleus accumbens, amygdala andhypothalamus degeneration were more marked in hexanucleotide repeat carriers than in *C9orf72*-negative ALS patients. The main finding of this study was the demonstration that limbic network degeneration is not unique to the *C9orf72* genotype, albeit disease burden was more marked in that group. Our multimodal data set of volumetric, cortical thickness and diffusivity variables also confirmed that interconnected grey and white matter components of entire networks were affected instead of selective focal pathological findings. This resonates with the recent shift from attributing clinical deficits to the degeneration of single structures, and highlights that clinical disability, be it motor, sensory, or neuropsychological is likely to be driven by the dysfunction of entire circuits with a multitude of grey and white matter components. This signals that care must be taken with direct correlations between clinical metrics and the integrity indices of single structures [66]. The underpinnings of domain-specific disability in ALS are best explored by connectomic approaches, network integrity assessments or, as alternatively demonstrated here, by comprehensively evaluating the integrity of cortical, subcortical and white matter components of specific networks.

### 4.1. Limbic system in Motor Neuron Disease

Both memory impairment and deficits in social cognition have a robust literature in ALS [36,67,68,69,70,71,72,73,74,75]. Limbic network dysfunction is relatively well recognized on clinical grounds, but there is a paucity of imaging studies specifically investigating limbic structures and the integrity of the Papez circuit [21,22,76]. There is a robust post-mortem literature describing limbic, mesial temporal and subcortical degeneration in ALS and in those with *C9orf72* expansion carriers [22,77,78]. Limbic network impairment has also been consistently detected in vivo using functional and structural imaging methods [79,80,81,82]. Some studies have previously specifically examined Papez circuit impairment in ALS, linking degenerative change to clinical performance [19,20,21], but genotype correlates have not been systematically assessed. There is also recognition that limbic regions may be affected in other non-ALS MNDs. While classically regarded as a “restricted” phenotype, with pathology relatively confined to motor regions [83], primary lateral sclerosis (PLS) is increasingly considered as a multi-system disease [84,85]. Subcortical grey matter degeneration has been recently described in PLS [86], which is consistent with recent reports describing neuropsychological deficits in PLS [87,88,89,90]. To a lesser extent, neuropsychological deficits have also been described in other MND phenotypes, such as spinal–bulbar muscular atrophy (SBMA) and progressive muscular atrophy (PMA) [91,92,93]. Interestingly, despite reports of cognitive deficits in poliomyelitis survivors [94,95], no widespread frontotemporal or subcortical change can be detected in polio survivors using multimodal imaging techniques [96].

### 4.2. Clinical Correlates of Hexanucleotide Carrier Status

The *C9orf72* repeat expansion is located within intron 1 of *C9orf72* at genomic position chr9:27,573,529-27,573,546 (GRCh38 coordinates), pointing to the three repeats that are found in the reference genome; the most common allele is, in fact, two repeats. Hexanucleotide repeat expansion means that the sequence GGGGCC in the *C9orf72* transcript, which is normally repeated two or three times (i.e., …GGGGCCGGGGCC…) is instead repeated hundreds of times. This expansion has no “function”, it is an error made during DNA replication. Its consequence in the cell is the formation of hairpins and G quadruplexes within the RNA transcript, which can cause aggregation of RNA and the sequestration of RNA binding proteins, as well as the formation of apparently toxic dipeptide repeats via repeat-associated non-AUG translation. The main clinical effect centers principally on neurodegeneration, manifesting in ALS and/or FTD. The identified anatomical patterns and limbic vulnerability in *C9orf72* carriers may be a consequence of the differential expression of *C9orf72* found in the neuronal subtypes of the limbic system, but future experiments using single-cell RNAseq in postmortem tissue are required to shed light on this. Soon after the discovery of hexanucleotide repeat expansions in ALS [97,98], a number of large clinical studies were published, describing marked cognitive and behavioural change in expansion carriers [77,78,99,100,101]. Subsequent imaging studies have also revealed considerable frontotemporal and subcortical degeneration in association with hexanucleotide carrier status [102,103,104,105,106], raising the question of whether this genotype accounts for most of the comorbid FTD cases we observe clinically. *C9orf72* carrier status does not explain all the comorbid FTD cases in ALS as severe subcortical and frontotemporal involvement may also be observed in sporadic and *C9orf72* negative cases [107]. In the past five years, family members of *C9orf72* ALS probands have increasingly been invited to participate in asymptomatic clinical and imaging studies as this genotype offers unrivalled opportunities to uncover the presymptomatic phase of ALS and FTD, and map propagation patterns in vivo. Presymptomatic imaging studies have consistently described subcortical, thalamic and white matter degeneration before symptom onset [103,108,109,110], but no validated indicators have yet been developed to predict estimated age of onset or the predominant clinical phenotype, i.e., will the hexanucleotide carrier develop ALS or FTD [103]. While different terminology is often used in Europe and North America (pre- versus a-symptomatic), there is an increasing consensus in splitting the presymptomatic phase of ALS into (1) premanifest and (2) prodromal phases [111,112]. This has gained practical relevance with the advent of promising antisense oligonucleotide (ASO) therapies [113,114] and opens a window of opportunity to intervene before irreversible degeneration ensues. The vast majority of presymptomatic studies in ALS were conducted in asymptomatic *C9orf72* carriers, but pioneering studies of asymptomatic SOD1 have also been published [115]. Many of these studies have confirmed extensive degenerative change many years before projected symptom onset, offering unique insights into the long and relatively arcane phase of disease biology preceding symptom onset. Some studies detected changes in very young individuals [108,116] several decades before typical symptom onset, and have suggested the reconsideration of these processes as developmental rather than neurodegenerative in nature [116,117]. The considerable disease burden detected long before symptom onset, during a phase of relatively preserved motor and cognitive function, raises questions about the inherent redundancy and resilience of key cerebral networks or potential adaptive processes to withstand these changes and continue to function [118,119]. While presymptomatic imaging and clinical studies of *C9orf72* provided invaluable insights into the patterns, timeline and dynamics of progressive degeneration, extreme care is needed not to extrapolate these changes observed in *C9orf72* to the presymptomatic phase of sporadic ALS or other genetic variants.

### 4.3. Methodological Considerations

Our study reveals a relative symmetry of limbic involvement with bilateral basal forebrain, subiculum and enthorinal cortex involvement in sporadic patients without *C9orf72* hexanucleotide repeats. Hexanucleotide repeat expansion carriers revealed a slightly different pattern of vulnerability, but also bilateral amygdala, hypothalamus, nucleus accumbens and posterior cingulate cortex and cingulum fiber involvement. The striking symmetry of these patterns suggests that, irrespective of site of onset, site of symptom onset and handedness, these structures exhibit a core vulnerability in ALS with bilateral degeneration. From a methods perspective, it is interesting that while fornix changes are readily captured by FA, AD and RD profiles, changes in the left cingulum in *C9orf72*-negative patients are only detected by AD, and not by FA and RD. Similarly, white matter integrity change in the right cingulum of *C9orf72* patients is detected by RD but not by FA. This highlights the relative drawbacks of only evaluating FA profiles in ALS, and the benefit of assessing multiple diffusivity metrics. Often, only FA is assessed as a composite marker of white matter integrity in imaging studies, but ALS studies have consistently demonstrated the importance of assessing RD and AD. RD has been traditionally considered a proxy of myelin integrity, and AD is sometimes thought of as an “axonal” marker [120,121], but the practical relevance of examining several diffusivity metrics in ALS lies in their different sensitivity in detecting, tracking and discriminating white matter profiles in various monitoring and diagnostic applications [122,123,124,125]. The presented analyses rely on routinely acquired T1-weighted raw data and a short-acquisition diffusion protocol. T1-weighted imaging is part of any clinical protocol, and is typically only visually inspected in the clinical setting. However, as demonstrated above, if data are acquired in 3D without slice gaps, T1w data can be comprehensively interrogated in a multitude of quantitative analysis streams such as subcortical structure segmentations, cortical thickness, cortical volume and cerebellar analyses (MT In Press). DTI can be readily interpreted in a variety of voxel-wise and tractographic approaches to assess white matter integrity. Novel Gaussian and non-Gaussian white matter techniques such as High Angular Resolution Diffusion Imaging (HARDI), neurite orientation dispersion and density imaging (NODDI) are thought to be particularly sensitive in detecting white matter changes, both in the presymptomatic and symptomatic patients [109,126,127]. While seldom used in clinical ALS care, magnetic resonance spectroscopy (MRS) has emerged as a powerful tool to detect both motor and extra-motor metabolic changes in ALS [128,129,130,131]. ALS also has a robust functional MRI (fMRI) literature [132,133], but the potential confounding effects of medications, hypoxia and underlying cerebrovascular changes are seldom acknowledged or discussed. More recently, several research groups implemented quantitative susceptibility (QSM) approaches to characterize both cortical and subcortical changes.

### 4.4. Clinical Relevance

Limbic network dysfunction and, more broadly, cognitive dysfunction, have very significant clinical ramifications as they not only impact on the management of the patient, but additional resources may need to be put in place for the optimal care of the patients, which may translate into additional caregiver burden and impact negatively on clinical trial participation. Large neuropsychology studies in ALS using comprehensive batteries of cognitive and behavioural tests have consistently highlighted executive and behavioural deficits in association with *C9orf72* status, as well as lower age of symptom onset [54]. While this present study is primarily a neuroimaging study and only cognitive screening tests were evaluated, poorer memory performance was detected in hexanucleotide repeat expansion carriers, contributing to a lower overall “ALS non-specific” score (Table 1). ECAS is primarily a screening tool, which evaluates both “ALS-specific” (language, verbal fluency, executive) and “ALS non-specific” (memory, visuospatial) domains [49]. Our regression models identified left entorhinal gyrus cortical thickness as a significant predictor (b = 1.526; *p* = 0.020) of memory performance in our larger (n=182) C9- cohort. The physiological role of the entorhinal cortex as network hub for memory processes is well established, and our data links cortical thickness alterations in this region to memory performance in ALS. Cognitive impairment affects compliance with assistive devices [34] in multiple ways; tolerating or synchronizing with non-invasive ventilation, learning and implementing secretion clearance techniques such as breath stacking or cough assist machines, driving motorized wheelchairs, maintaining feeding tube hygiene, using electronic devices with predictive texting and capitalizing on speech banking [34]. Cognitive deficits are also thought to affect engagement with multidisciplinary interventions, fall prevention, adherence to medications and hamper both advance care planning and rehabilitation efforts. Neuropsychological deficits in ALS have been consistently linked to increased caregiver burden, and are also recognized as a negative prognostic indicator with a faster rate of decline and adverse survival ramifications [35]. Behavioural impairment, especially early in the course of the disease, may be mistaken for psychiatric conditions. Apathy, which is increasingly recognized as a relatively common sequela of ALS, may impact on clinic attendance and motivation to participate in research and clinical trials [134,135]. Apathy has been previously linked to nucleus accumbens degeneration in ALS, and it not only impacts on daily activities but may also impact on research participation. It is likely that patients with significant cognitive deficits, and apathy in particular, are underrepresented in academic research studies. This may be particularly true for non-therapeutic (non-pharmacological) biomarker studies [136], such as neuroimaging studies where attendance at a dedicated imaging center may be particularly taxing. As demonstrated by the above examples, cognitive and behavioural deficits in ALS have a number of grave practical ramifications for the management of ALS, making the study of the underlying processes a worthy and clinically relevant pursuit.

### 4.5. Diagnostic and Monitoring Applications

Neuroimaging in ALS has gained considerable momentum in recent years and departed from focal structural analyses to comprehensively evaluate connectivity, and metabolic and functional alterations in specific phenotypes and genotypes. Large descriptive studies relying on group comparisons have been gradually superseded by studies interpreting single data sets from individual patients using either large normative data sets [137,138] or machine learning algorithms [139,140,141]. A key step in the development of effect machine learning algorithm is the selection of discriminating features. While motor regions and motor tracts seem obvious features, recent studies have demonstrated the diagnostic utility of evaluating subcortical and extra-motor radiology metrics as well [123,142]. The radiological heterogeneity of ALS was initially explored by studies stratifying their patients based on clinical criteria such as disease-onset, comorbid dementia or genotype status [17], and recent studies have explored naturally occurring clusters without imposing clinical categorization on the data [143,144,145]. These studies have consistently identified homogenous sub-cohorts of patients with more extensive frontotemporal change [144,145] without using accessory clinical or neuropsychological information. The characterization of limbic pathology and the genotype-dependent involvement of these structures would therefore indicate that the quantitative assessment of these structures may have a role in future ML applications. MRI-derived integrity metrics have also been extensively evaluated in large multi-timepoint longitudinal studies to assess rate of decline, progression rates and propagation patterns. Some metrics have proved particularly sensitive in detecting subtle changes over relatively short follow-up intervals, suggesting a putative monitoring role or as potential outcome measures in clinical trials [146,147,148,149].

### 4.6. Study Limitations

This study is not without limitations. Despite our efforts to interrogate a multiparametric data set with complementary volumetric, cortical thickness and diffusivity metrics, we have not evaluated functional MRI data in this study. While our analyses compellingly demonstrate considerable limbic system pathology in both patient cohort, we have no supporting post-mortem data to examine the neuropathological and proteinopathic underpinnings of these radiological changes. It would be of particular interest to assess the pTDP-43 burden in these nuclei. Similarly, we describe white matter changes in both the cingulum and fornix, based on diffusion data, but it would be of considerable interest to assess axonal, myelin-related changes histopathologically. One of the biggest shortcomings of this study stems from its cross-sectional design, which precludes the characterization of the timeline of limbic changes. As both patient cohorts have a considerable symptom duration (Table 1), the question remains whether the notably symmetric bilateral involvement is a reflection of long symptom duration or an aspect of the fundamental vulnerability of limbic structures in ALS. Ultimately, only longitudinal and presymptomatic studies can characterize the exact chronology of limbic and motor changes, i.e., which one develops first. Recent presymptomatic studies seem to detect thalamic and subcortical pathology before motor changes become detectable [110], but pathological staging systems suggest otherwise [150]. Finally, while standard clinical questionnaires, rating scales and cognitive screening tests have been administered, more detailed neuropsychological testing would have been desirable and would have provided additional opportunities to map cognitive deficits to radiological changes. Notwithstanding these limitations, our study demonstrates bilateral limbic system pathology in both sporadic patients with ALS and in hexanucleotide repeat expansion carriers.

## 5. Conclusions

We demonstrated considerable limbic system and Papez circuit degeneration in both hexanucleotide repeat expansion carriers and patients who tested negative for *C9orf72*. Our results highlight that mesial temporal and parasagittal subcortical degeneration is not unique to *C9orf72* carriers. Our radiological findings are consistent with previous neuropsychological observations and highlight the importance of comprehensive cognitive testing in ALS, irrespective of the underlying genotype. Cognitive impairment in ALS has widespread practical implications, including compliance with assistive devices and participation in clinical trials, has been associated with increased caregiver burden and is widely regarded as an adverse prognostic indicator.

## Figures and Tables

**Table 1:** The demographic and clinical profile of study participants.

Study Groups	C9-ALS (*n* = 182)	C9+ALS (*n* = 22)	HC (*n* = 111)	*p*-Value
Age (years)	61.57 ± 12.28	58.00 ± 8.98	59.55 ± 10.81	0.195
Sex (M/F)	120/62	14/8	54/57	0.013
Education (years)	13.76 ± 3.33	13.95 ± 3.35	13.03 ± 3.60	0.170
Handedness (R/L)	174/8	19/3	98/13	0.040
Site onset (S/B)	160/22	20/2	n/a	0.680
Symptom duration (months)	17.12 ± 5.78	16.18 ± 6.13	n/a	0.239
ALSFRS-R	38.72 ± 5.67	37.91 ± 6.85	n/a	0.268
ECAS-Total Score	104.67 ± 15.36	100.27 ± 19.18	n/a	0.263
ECAS-ALS Specific score	76.99 ± 12.51	75.47 ± 15.77	n/a	0.665
ECAS-ALS Non-specific score	27.68 ± 5.46	24.80 ± 5.54	n/a	0.040
ECAS-Language	24.24 ± 3.94	25.27 ± 3.10	n/a	0.306
ECAS-Verbal Fluency	16.66 ± 5.04	17.13 ± 5.74	n/a	0.734
ECAS-Executive Functions	36.10 ± 7.26	33.07 ± 8.67	n/a	0.126
ECAS-Memory	16.24 ± 4.42	13.93 ± 5.32	n/a	0.047
ECAS-Visuospatial Functions	11.44 ± 2.66	10.87 ± 1.06	n/a	0.396

**Table 2:** Volumes of limbic structures (mm^3^) in healthy controls (HC), *C9orf72*-negative patients with ALS (C9-ALS) and *C9orf72*-positive patients with ALS (C9+ALS). Estimated marginal means (EMM) and standard errors (SE) were adjusted for age, sex, handedness and total intracranial volume (TIV). Significant intergroup differences at *p* < 0.05 after Bonferroni correction for multiple comparisons are highlighted in bold print.

Limbic Structure	Study Group	Descriptive Values		Statistics			
		EMM	Standard Error	Univariate F, *p*-Value	C9-ALS vs. HC	C9+ALS vs. HC	C9- vs. C9+
**Amygdala L**	C9-ALS	1508.243	18.000	F = 8.570; *p* < 0.001	0.070	**<0.001**	**0.012**
	C9+ALS	1349.043	51.753				
	HC	1575.883	23.153				
**Basal Forebrain L**	C9-ALS	306.179	2.901	F = 4.219; *p* = 0.016	**0.049**	0.067	0.866
	C9+ALS	296.769	8.342				
	HC	317.749	3.732				
**Fornix L**	C9-ALS	514.843	4.373	F = 2.326; *p* = 0.099	0.795	0.108	0.353
	C9+ALS	493.907	12.573				
	HC	522.893	5.625				
**Hypothalamus L**	C9-ALS	463.698	3.482	F = 6.096; *p* = 0.003	1.000	**0.002**	**0.003**
	C9+ALS	428.811	10.011				
	HC	466.398	4.479				
**Mammillary Body L**	C9-ALS	52.900	0.572	F = 0.028; *p* = 0.973	1.000	1.000	1.000
	C9+ALS	52.761	1.645				
	HC	52.680	0.736				
**Nucleus Accumbens L**	C9-ALS	394.490	5.605	F = 7.448; *p* < 0.001	0.174	**<0.001**	**0.013**
	C9+ALS	345.218	16.116				
	HC	412.086	7.210				
**Septal Nucleus L**	C9-ALS	121.623	1.012	F = 0.017; *p* = 0.983	1.000	1.000	1.000
	C9+ALS	121.550	2.909				
	HC	121.320	1.301				
**Subiculum L**	C9-ALS	423.627	4.267	F = 6.982; *p* = 0.001	**0.016**	**0.004**	0.213
	C9+ALS	400.029	12.268				
	HC	443.416	5.489				
**Amygdala R**	C9-ALS	1737.101	19.945	F = 7.805; *p* < 0.001	**0.047**	**<0.001**	**0.035**
	C9+ALS	1582.775	57.346				
	HC	1816.935	26.655				
**Basal Forebrain R**	C9-ALS	324.076	3.131	F = 10.822; *p* < 0.001	**<0.001**	**0.001**	0.392
	C9+ALS	309.597	9.003				
	HC	344.626	4.028				
**Fornix R**	C9-ALS	520.230	4.416	F = 2.493; *p* = 0.084	0.713	0.092	0.331
	C9+ALS	498.640	12.698				
	HC	528.845	5.681				
**Hypothalamus R**	C9-ALS	466.699	3.323	F = 5.742; *p* = 0.004	1.000	**0.003**	**0.006**
	C9+ALS	435.261	9.554				
	HC	470.453	4.274				
**Mammillary Body R**	C9-ALS	55.373	0.570	F = 0.532; *p* = 0.588	1.000	1.000	1.000
	C9+ALS	56.408	1.638				
	HC	54.718	0.733				
**Nucleus Accumbens R**	C9-ALS	388.658	5.257	F = 8.121; *p* < 0.001	0.370	**<0.001**	**0.003**
	C9+ALS	335.512	15.116				
	HC	402.058	6.762				
**Septal Nucleus R**	C9-ALS	116.517	0.987	F = 0.134; *p* = 0.875	1.000	1.000	1.000
	C9+ALS	117.504	2.839				
	HC	117.266	1.270				
**Subiculum R**	C9-ALS	415.900	3.907	F = 4.151; *p* = 0.017	**0.036**	0.104	1.000
	C9+ALS	406.041	11.234				
	HC	432.160	5.026				

**Table 3:** Cortical thickness (mm) profiles in healthy controls (HC), C9orf72-negative patients with ALS (C9-ALS) and C9orf72-positive patients with ALS (C9+ALS). Estimated marginal means (EMM) and standard errors (SE) were adjusted for age, sex and handedness. Significant intergroup differences at *p* < 0.05 after Bonferroni correction for multiple comparisons are highlighted in bold print.

Limbic Structure	Study Group	Descriptive Values		Statistics			
		EMM	Standard Error	Univariate F, *p*-Value	C9-ALS vs. HC	C9+ALS vs. HC	C9- vs. C9+
**ACC-caudal L**	C9-ALS	2.589	0.017	F = 2.491; *p* = 0.084	0.915	0.084	0.263
	C9+ALS	2.498	0.050				
	HC	2.619	0.022				
**ACC-rostral L**	C9-ALS	2.657	0.015	F = 10.563; *p* < 0.001	**0.010**	**<0.001**	**0.016**
	C9+ALS	2.530	0.043				
	HC	2.730	0.019				
**Entorhinal cortex L**	C9-ALS	3.255	0.028	F = 6.227; *p* = 0.002	**0.006**	**0.035**	1.000
	C9+ALS	3.178	0.079				
	HC	3.398	0.035				
**Parahippocampal gyrus L**	C9-ALS	2.749	0.021	F = 2.152; *p* = 0.118	0.121	1.000	1.000
	C9+ALS	2.763	0.061				
	HC	2.822	0.027				
**PCC L**	C9-ALS	2.447	0.012	F = 6.718; *p* = 0.001	0.376	**<0.001**	**0.011**
	C9+ALS	2.343	0.034				
	HC	2.477	0.015				
**ACC-caudal R**	C9-ALS	2.436	0.016	F = 7.523; *p* < 0.001	**<0.001**	0.076	1.000
	C9+ALS	2.422	0.016				
	HC	2.534	0.021				
**ACC-rostral R**	C9-ALS	2.777	0.015	F = 2.804; *p* = 0.062	0.073	0.500	1.000
	C9+ALS	2.768	0.015				
	HC	2.833	0.019				
**Entorhinal cortex R**	C9-ALS	3.319	0.028	F = 3.881; *p* = 0.022	**0.022**	0.380	1.000
	C9+ALS	3.309	0.079				
	HC	3.442	0.036				
**Parahippocampal gyrus R**	C9-ALS	2.734	0.017	F = 0.963; *p* = 0.383	1.000	0.569	1.000
	C9+ALS	2.687	0.049				
	HC	2.758	0.022				
**PCC R**	C9-ALS	2.418	0.013	F = 8.700; *p* < 0.001	0.217	**<0.001**	**0.003**
	C9+ALS	2.291	0.037				
	HC	2.456	0.016				

**Table 4:** White matter diffusivity profiles in healthy controls (HC), *C9orf72*-negative patients with ALS (C9-ALS) and *C9orf72*-positive patients with ALS (C9+ALS). Estimated marginal means (EMM) and standard errors are adjusted for age, sex and handedness. Significant intergroup differences at *p* < 0.05 after Bonferroni correction for multiple comparisons are highlighted in bold print.

Limbic Structure	Study Group	Descriptive Values		Statistics			
		EMM	Standard Error	Univariate F, *p*-Value	C9-ALS vs. HC	C9+ALS vs. HC	C9- vs. C9+
**Cingulum-FA L**	C9-ALS	0.505	0.004	F = 8.079; *p* < 0.001	1.000	**<0.001**	**<0.001**
	C9+ALS	0.461	0.011				
	HC	0.510	0.005				
**Cingulum-AD L** **(×10^−3^)**	C9-ALS	1.259	0.006	F = 4.720; *p* = 0.010	**0.012**	0.180	1.000
	C9+ALS	1.265	0.017				
	HC	1.231	0.008				
**Cingulum-RD L** **(×10^−3^)**	C9-ALS	0.550	0.006	F = 10.938; *p* < 0.001	0.213	**<0.001**	**<0.001**
	C9+ALS	0.614	0.016				
	HC	0.533	0.007				
**Cingulum-FA R**	C9-ALS	0.445	0.004	F = 10.733; *p* < 0.001	0.098	**<0.001**	**0.001**
	C9+ALS	0.404	0.011				
	HC	0.458	0.005				
**Cingulum-AD R** **(×10^−3^)**	C9-ALS	1.202	0.006	F = 3.331; *p* = 0.037	0.177	0.073	0.581
	C9+ALS	1.226	0.018				
	HC	1.183	0.008				
**Cingulum-RD R** **(×10^−3^)**	C9-ALS	0.592	0.006	F = 13.204; *p* < 0.001	**0.029**	**<0.001**	**<0.001**
	C9+ALS	0.658	0.016				
	HC	0.567	0.007				
**Fornix-FA**	C9-ALS	0.275	0.002	F = 20.430; *p* < 0.001	**0.013**	**<0.001**	**<0.001**
	C9+ALS	0.239	0.007				
	HC	0.286	0.003				
**Fornix-AD** **(×10^−3^)**	C9-ALS	1.869	0.018	F = 26.935; *p* < 0.001	**<0.001**	**<0.001**	**<0.001**
	C9+ALS	2.141	0.053				
	HC	1.737	0.024				
**Fornix-RD** **(×10^−3^)**	C9-ALS	1.243	0.016	F = 28.212; *p* < 0.001	**<0.001**	**<0.001**	**<0.001**
	C9+ALS	1.500	0.016				
	HC	1.131	0.021				

## Data Availability

Personal Patient Data is not publicly available due to departmental policies.

## Glossary

**AAL**: Automated Anatomical Labeling (AAL) atlas, **AD**: axial diffusivity, **ALS**: amyotrophic lateral sclerosis, **ALSod**: ALS online database, **ANOVA**: analysis of variance (ANOVA), **ASO**: antisense oligonucleotide, **BOLD**: blood-oxygen-level-dependent (BOLD) signal, **C9+**: ALS patients with GGGGCC hexanucleotide repeat expansion in C9orf72, **C9-**: ALS patients without GGGGCC hexanucleotide repeat expansion in C9orf72, ***C9orf72***: chromosome 9 open reading frame 72, **CC**: Corpus callosum, **CI**: confidence interval, **CT**: cortical thickness, **CSD**: constrained spherical deconvolution, **CST**: Corticospinal tract, **DOFs**: degrees of freedom, **DTI**—diffusion tensor imaging, **DWI**: diffusion-weighted imaging, **EMG**: electromyography, **EMM**: estimated marginal mean, **EPI**: echo-planar imaging, **FA**: fractional anisotropy, **FC**: functional connectivity, **fMRI**: functional MRI, **FLAIR**: fluid-attenuated inversion recovery, **fODF**: fibre orientation distribution function, **FOV**: field of view, **FSL**: FMRIB’s Software Library, **FTD**: frontotemporal dementia, **FTLD**: Frontotemporal lobar degeneration, **FWE**: familywise error, **GM**: gray matter, **HARDI**: High Angular Resolution Diffusion Imaging, **HC**: healthy control, **HD**: Huntington’s disease, **ICA-AROMA**: Automatic Removal Of Motion Artifacts, **IR-SPGR**: inversion recovery prepared spoiled gradient recalled echo, **IQR**: interquartile range, **LAS**: Local Adaptive Segmentation, **LH**: left hemisphere, **Lt**: Left, **LMN**: lower motor neuron, **M1**: Primary motor cortex, **MANCOVA**: multivariate analyses of covariance, **ML**: machine-learning, **MND**: Motor neuron disease, **MNI**: Montreal Neurological Institute, **MNI152**: Montreal Neurological Institute 152 standard space, **MRI**: magnetic resonance imaging, **MRS**: MR spectroscopy, **NISALS**: Neuroimaging Society in ALS, **NIV**: non-invasive ventilation, **NODDI**: neurite orientation dispersion and density imaging, ***p*_adj_**: adjusted *p*-value, **PBA**: Pseudobulbar affect, **PCR**: polymerase chain reaction, **PD**: Parkinson’s disease, **PMA**: progressive muscular atrophy (PMA), **PMC**: primary motor cortex, **QC**: quality control, **RH**: right hemisphere, **Rt**: right, **RD**: Radial diffusivity, **ROI**: region of interest, **rs-fMRI**: resting-state functional MRI, **SBMA**: spinal-bulbar muscular atrophy, **SC**: structural connectivity, **SD**: standard deviation, **SE-EPI**: spin echo planar imaging, **SENSE**: sensitivity Encoding, **SPIR**: spectral presaturation with inversion recovery, **T**: Tesla, **T1w**: T1-weighted imaging, **TCV**: total cerebellar volume, **TDI**: Track Density Imaging, **TE**: echo time, **TI**: inversion time, **TIV**: total intracranial volume, **Tukey HSD**: Tukey’s Honest Significant Difference, **TR**: repetition time, **UMN**: upper motor neuron, **VR**: voxel resolution, **WM**: white matter.
